# Non-coding RNAs in tumor-educated platelets: emerging roles in cancer progression, biomarker discovery, and therapeutic targeting

**DOI:** 10.3389/fonc.2026.1951597

**Published:** 2026-09-22

**Authors:** Yaning Liu, Yucheng Zhang, Dongni Li, Jian Zhao, Mohan Liu, Yali Wang

**Affiliations:** 1 Department of Blood Transfusion, China–Japan Union Hospital of Jilin University, Changchun, Jilin, China; 2 Scientific Research Center, China–Japan Union Hospital of Jilin University, Changchun, Jilin, China; 3 Department of Clinical Medicine, China-Japan Union Hospital of Jilin University, Changchun, Jilin, China; 4 Department of Clinical Medicine, The Third Norman Bethune Clinical College of Jilin University, Changchun, Jilin, China

**Keywords:** cancer biomarkers, circular RNAs, liquid biopsy, long non-coding RNAs, microRNAs, non-coding RNAs, tumor progression, tumor-educated platelets

## Abstract

Non-coding RNAs (ncRNAs) in tumor-educated platelets (TEPs) play an important role in cancer progression. Tumor cells can “educate” platelets, thereby enabling them to participate in multiple stages of tumor development and progression. The ncRNAs carried by TEPs, including microRNAs (miRNAs), circular RNAs (circRNAs), and long non-coding RNAs (lncRNAs), may regulate tumor cell behavior and influence processes such as proliferation, invasion, metastasis, immune regulation, and angiogenesis. Alterations in the expression profiles of these ncRNAs may provide potential biomarkers for early cancer diagnosis, prognosis assessment and treatment monitoring. They may also offer new opportunities for therapeutic intervention. This review summarizes the mechanisms of platelet education by tumor cells, the roles of TEPs in cancer progression, and the functional significance of ncRNAs in TEPs, with particular focus on their contributions to tumor development and progression, their biomarker potential, and their potential as therapeutic targets.

## 1 Introduction

In the rapidly evolving field of cancer research, tumor-educated platelets (TEPs) have emerged as an important focus for understanding the dynamic relationship between tumor cells and platelets. Tumor progression is not merely the result of unchecked cell proliferation, but is also shaped by dynamic interactions between tumor cells and their surrounding microenvironment (1, 2).

Platelets, highly abundant anucleate cellular components of blood, are traditionally recognized for their roles in hemostasis and thrombosis. However, recent research has revealed that platelets also actively contribute to shaping the tumor microenvironment (TME). Tumor-derived cytokines, metabolites, and microenvironmental signals remotely regulate megakaryopoiesis, thrombopoiesis, and the selective packaging of molecular cargo into platelets. Meanwhile, circulating platelets actively take up tumor-released RNAs, proteins, DNA, and extracellular vesicles, thereby acquiring tumor-specific molecular signatures. Notably, anucleate platelets possess functional spliceosomes and RNA processing machinery, allowing tumor-induced RNA splicing modifications and transcriptomic remodeling (3, 4). These TEPs function both as dynamic circulating biomarkers for tumor burden assessment, molecular classification, and treatment response monitoring, and as active participants in tumor progression through the secretion of bioactive molecules and vesicles (5, 6). Critically, platelet education is distinct from non-specific platelet activation; it entails tumor-driven molecular and functional reprogramming rather than mere activation alone (7).

Compared to conventional platelet-derived biomarkers, platelet ncRNAs may offer several potential advantages, including relatively high molecular stability, cancer-associated expression pattern, and compatibility with sensitive and multiplexed molecular detection (8). In addition, because ncRNAs can act as regulatory molecules, their alterations may provide both biomarker information and mechanistic insight into tumor–platelet communication (9, 10). These characteristics render platelet-associated ncRNAs particularly attractive candidates for liquid biopsy and therapeutic exploration.

Recent reviews have concentrated on the crosstalk between platelets and tumors, the mechanisms of platelet education, and the applications of TEPs in liquid biopsy (11). However, the specific contributions of platelet-associated ncRNAs remain inadequately integrated. In particular, important unresolved questions include how platelets are educated by tumors, how this education alters platelet molecular cargo, and how ncRNAs within TEPs may contribute to cancer progression, diagnosis, and therapeutic development. Therefore, this review first outlines the major mechanisms of tumor-mediated platelet education, then discusses the biological roles of TEPs in cancer progression, and finally summarizes the mechanisms of platelet miRNAs, circRNAs, and lncRNAs in tumor progression, with an emphasis on their potential as liquid-biopsy biomarkers and therapeutic targets. Unlike reviews focused primarily on general TEP biology, platelet–tumor crosstalk, or liquid-biopsy applications, this review adopts an ncRNA-centered framework that systematically links tumor-mediated platelet education and ncRNA remodeling with the functional effects of TEP-associated ncRNAs on tumor progression and their diagnostic and therapeutic translational potential.

## 2 Tumor-mediated platelet education

### 2.1 Remote tumor-mediated remodeling of the megakaryocyte–platelet production axis

Tumor-mediated education of platelets can occur during megakaryocyte differentiation and thrombopoiesis. Evidence indicates that tumors can remotely regulate megakaryocytes in the bone marrow and possibly the lung through cytokines, metabolites, and extracellular vesicles, thereby altering platelet count, volume, RNA profiles, and protein composition (12). Tumor-associated thrombocytosis represents a key manifestation of such remote regulation. Tumor cells and the tumor microenvironment can release IL-6, which enters the circulation and stimulates the liver to produce thrombopoietin (TPO). TPO then acts on the c-MPL receptor on the surface of megakaryocytes, promoting megakaryocyte proliferation, maturation, and platelet release, ultimately leading to paraneoplastic thrombocytosis (12, 13).

In addition to the IL-6–TPO axis, tumor-derived PF4 and VEGF, along with other factors, may also participate in the regulation of platelet production (**Figure 1**). PF4 can promote platelet production and support lung cancer growth (14). VEGF is not only involved in tumor angiogenesis but may also influence platelet production by modulating the bone marrow vascular microenvironment and affecting megakaryocyte maturation (15). Tumor metabolites may also participate in the upstream processes of TEP formation. Tumor cells can produce kynurenine (Kyn) through tryptophan metabolism. Once taken up by myeloid progenitors, Kyn activates the AhR–RUNX1 axis, driving megakaryocyte–erythroid progenitors toward megakaryocytic differentiation and thereby promoting platelet production. This finding indicates that tumors can alter lineage commitment in platelet-originating cells through metabolic reprogramming, which may further affect the molecular composition and functional state of newly generated platelets (16).

**Figure 1:**
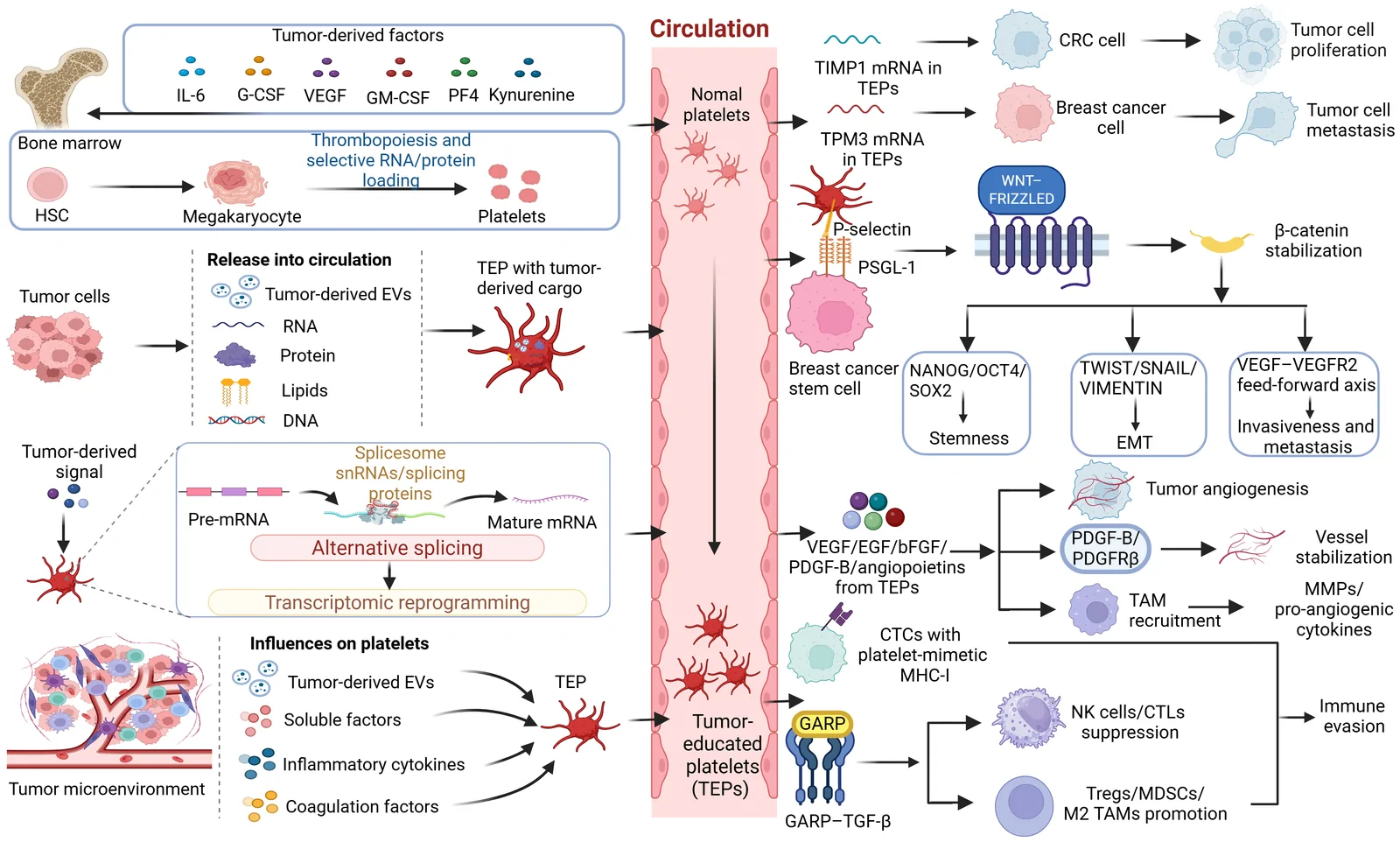
Platelet education by tumor cells and the mechanisms of tumor-educated platelets in cancer. Tumor cells and the tumor microenvironment release systemic factors, including interleukin-6 (IL-6), granulocyte colony-stimulating factor (G-CSF), vascular endothelial growth factor (VEGF), granulocyte-macrophage colony-stimulating factor (GM-CSF), platelet factor 4 (PF4), and kynurenine. These signals act on hematopoietic stem/progenitor cells (HSPCs) and megakaryocytes in the bone marrow, regulating megakaryocyte differentiation, maturation, thrombopoiesis, the selective loading of RNAs and proteins into nascent platelets. Tumor cells release extracellular vesicles, RNAs, ncRNAs, proteins, lipids, and DNA into the circulation. These components can be internalized and stored by platelets, allowing platelets to acquire tumor-associated molecular information. Platelets retain pre-mRNAs, spliceosomes, snRNAs, and associated splicing proteins. Tumor-derived signals may trigger alternative splicing and transcriptomic reprogramming, thereby modifying platelet RNA cargo and functional status. Tumor-derived extracellular vesicles, soluble factors, inflammatory cytokines, coagulation factors, and other tumor microenvironmental signals collectively influence platelet phenotype and function. Through these mechanisms, naïve platelets are educated into tumor-educated platelets (TEPs). TIMP mRNA in TEPs promotes tumor cell proliferation whereas TPM3 mRNA in TEPs promote tumor cell metastasis. Platelet P-selectin binds to PSGL-1 on breast cancer stem cells, activates WNT-FRIZZLED signaling and β-catenin stabilization, and further induces NANOG/OCT4/SOX2-mediated stemness, TWIST/SNAIL/VIMENTIN-associated epithelial-mesenchymal transition (EMT), and the VEGF–VEGFR2 feed-forward axis-associated invasiveness and metastasis. TEP-derived VEGF, EGF, bFGF, PDGF-B, and angiopoietins can promote tumor angiogenesis, facilitate vessel stabilization via the PDGF−B/PDGFRβ signaling axis, and drive the recruitment of tumor-associated macrophages (TAMs), further amplifying the release of matrix metalloproteinases (MMPs) and pro-angiogenic cytokines. GARP−TGF−β signaling, which mediates the suppression of NK cells and cytotoxic T lymphocytes (CTLs) and promotes the generation of regulatory T cells (Tregs), myeloid−derived suppressor cells (MDSCs), and M2−polarized TAMs, together with circulating tumor cells (CTCs) that acquire a platelet−mimetic MHC−I phenotype, collectively shape an immune−evasive tumor microenvironment.

However, cancer patients may also develop thrombocytopenia as a result of various factors, including chemotherapy, disseminated intravascular coagulation (DIC), bone marrow infiltration, and tumor-derived inhibitory factors (17). Therefore, tumor-associated changes in platelet production are heterogeneous and may vary according to tumor type, disease stage, and treatment context. Additionally, tumors mediate the reprogramming of platelet expression profiles by modulating megakaryocyte differentiation and molecular characteristics. In spontaneous murine breast cancer models, primary tumors induce disrupted polyploidization and a pro-inflammatory transcriptional phenotype in bone marrow megakaryocytes. This is characterized by upregulated mRNA and protein levels of pro-inflammatory genes, including S100a8, S100a9, and Lcn2. These molecular alterations are transmitted to platelet progeny during thrombopoiesis, ultimately leading to modified proteomic profiles and enhanced pro-metastatic functions in circulating platelets (18).

Collectively, these findings indicate that tumor education can begin before platelets enter the circulation. This upstream remodeling provides an important basis for TEP formation.

### 2.2 Uptake of tumor-derived RNA, proteins, DNA, and extracellular vesicles

Although mature platelets are anucleate, they possess the ability to take up, store, and transport exogenous molecules. Tumor cells can release RNAs, proteins, lipids, DNA, and extracellular vesicles (19) (**Figure 1**). After entering the circulation, these tumor-derived components may be internalized by platelets, enabling platelets to acquire tumor-associated molecular information. A significant characteristic of TEPs, in contrast to normal platelets, is their enrichment with tumor-associated molecular cargo, which includes mutant transcripts, fusion gene transcripts, tumor-associated non-coding RNAs, tumor-derived DNA, and tumor-associated proteins (20, 21).

The uptake of tumor-derived RNA by platelets represents one of the classical lines of evidence in TEP research. Nilsson et al. demonstrated that tumor-derived RNA biomarkers, such as glioma-associated EGFRvIII, can be detected in platelets, indicating that platelets have the capacity to enrich tumor-derived transcripts and serve as a source of information for liquid biopsy (22). This study provides important theoretical validation for the transfer of tumor-derived RNAs into platelets. However, the patient cohort was relatively small and limited to a small set of specific tumor-associated transcripts. Furthermore, the functional activity of these transferred tumor-derived RNAs within platelets remains inadequately elucidated. Tumor-derived proteins can also be taken up and stored by platelets. Kerr et al. found that these proteins are stored in platelet granules and participate in the formation of the pre-metastatic bone microenvironment (23). Additionally, increased levels of VEGF, PF4, and PDGF have been observed in platelets from patients with colorectal cancer, suggesting that platelets may alter their protein cargo through endocytosis or selective sequestration (24). Notably, alterations in platelet proteins are not entirely consistent across different tumor models. For instance, TGF-β1 and MMP-1 demonstrate divergent expression patterns in prostate cancer and melanoma models, suggesting that the uptake and enrichment of tumor-derived proteins by platelets may exhibit significant tumor-type dependence (23). Therefore, its generalizability across distinct tumor types and patient populations requires further validation.

Tumor-derived extracellular vesicles serve as important mediators of platelet education. Dudiki et al. demonstrated that extracellular vesicles (EVs) derived from aggressive prostate cancer cells can be rapidly taken up by platelets, a process mediated by the EV membrane protein CD63. The uptake of EVs can lead to the accumulation of tumor-specific RNA, such as lncRNAs PCA3 and ENST00000501280 in platelets and induce non-canonical platelet activation and thrombosis through a CD63–RPTPα-related pathway (25). In addition to RNAs and proteins, platelets can also sequester extracellular DNA. Murphy et al. showed that platelets can capture endogenous cell-free DNA (cfDNA), including tumor-derived DNA and free fetal DNA (20).

These findings support that the capacity of platelets internalizine tumor-associated RNAs, proteins, DNA, and EVs. However, the level of evidence varies among different classes of molecular cargo. Future studies are necessary to ascertain whether these mechanisms are applicable across various tumor types and to clarify whether the molecular cargo internalized by platelets can further alter their biological functions.

### 2.3 Tumor signal-induced platelet RNA splicing and transcriptomic reprogramming

The landmark study by Denis et al. demonstrated that platelets harbor functional spliceosomes, which include small nuclear RNAs, splicing proteins, and endogenous pre-mRNAs. In addition, signal-dependent pre-mRNA splicing can occur in response to external stimulation (26). Tumor-associated signals acting as exogenous stimuli may reshape the platelet RNA profile by altering the splicing and stability of pre-existing pre-mRNAs in platelets (**Figure 1**). Platelets can retain pre-mRNAs derived from megakaryocytes and splice them into mature mRNAs following external stimulation. Factors secreted by tumors, tumor-derived vesicles, inflammatory cytokines, and coagulation-related signals may all affect this process, ultimately leading to the formation of a TEP transcriptome characterized by tumor-associated features (27).

Ding et al. further elucidated this mechanism, noting that platelets retain functional spliceosomes composed of U1, U2, U4, U5, and U6 small nuclear RNAs (snRNAs) and their associated proteins. U1 and U2 recognize the 5′ splice site and branch point, respectively, while the U4/U6·U5 tri-snRNP subsequently drives spliceosome assembly and catalytic rearrangement (28). Therefore, changes in snRNA abundance or spliceosomal composition can directly affect exon inclusion, exon skipping, intron retention, and the generation of distinct transcript isoforms. Tumors may reshape this splicing program at two levels. During platelet biogenesis, tumor-associated systemic signals may alter the selective sorting of pre-mRNAs, snRNAs, and splicing proteins from megakaryocytes into nascent platelets (18). Following their release into circulation, platelets may also internalize tumor-derived extracellular vesicles and their snRNA cargo, thereby further modifying spliceosomal composition and function (28). Early studies showed that the platelet RNA expression profile of patients with metastatic lung cancer differs significantly from that of healthy individuals, with approximately 200 RNA transcripts altered and differential splicing of SIRT2 observed (29). These findings indicate that tumors can systemically alter platelet RNA profiles.

However, direct evidence linking specific tumor-derived signals to defined platelet splicing events remains limited. Further studies are required to clarify how tumor signals regulate spliceosome activity, transcript isoform generation, and ncRNA profiles in TEPs.

TEP molecular signatures do not arise solely from the direct transfer of RNA from tumor cells to platelets. The tumor microenvironment also contains various cell types, including immune cells and endothelial cells, which can modify the transcriptome and proteome of platelets (30, 31). Therefore, TEPs should be regarded as a tumor-associated molecular state of platelets shaped by both direct tumor-derived signals and broader systemic influences (**Figure 1**).

Using deconvolution analysis of the TEP transcriptome, Karp et al. suggested that glioblastoma-associated inflammation may drive a subset of platelets into a highly activated state (7). These activated platelets are likely to interact more readily with monocytes and T cells within the tumor microenvironment, thereby acquiring their transcripts either actively or passively. These exogenous inflammatory signals may interact with the intrinsic activation-associated transcriptomic program of platelets, ultimately resulting in a tumor-associated TEP signature. Furthermore, Karp et al. identified that WFDC1 is predominantly found in a small subset of activated platelets associated with glioblastoma. Given that WFDC1 is expressed in endothelial cells associated with the blood-brain barrier, activated platelets may interact with tumor vascular endothelium and take up endothelial-derived transcripts following glioma-induced vascular barrier breakdown (7).

Overall, TEPs represent a specific platelet state formed through the combined effects of the tumor microenvironment, inflammatory cells, tumor-derived EVs, and endogenous platelet RNA processing. Among the molecular components remodeled through these processes, ncRNAs are particularly important because they may act not only as tumor-associated signatures but also as functional regulators of platelet–tumor communication.

## 3 Specific roles of tumor-educated platelets in cancer progression

Tumor-mediated platelet education is a multifaceted process involving remote remodeling of the megakaryocyte–platelet axis, uptake of tumor-derived molecular cargo, and signal-dependent RNA processing and transcriptomic reprogramming within platelets. Together, these processes remodel the molecular cargo and functional landscape of platelets, enabling TEPs to carry tumor-specific molecular signatures and potentially influence cancer progression.

### 3.1 TEPs as carriers of tumor molecular information

Compared with normal platelets, a major distinguishing feature of TEPs is that their molecular cargo is systemically reshaped by tumors, allowing them to carry tumor-associated RNAs, proteins, DNA, splicing events, and non-coding RNAs. Under the continuous influence of tumor cells, tumor-derived extracellular vesicles, and signals from the tumor microenvironment, TEPs develop tumor-associated molecular profiles (32).

RNA-seq studies have shown that TEP RNA profiles can accurately distinguish cancer patients from healthy individuals and further classify different tumor types as well as cancer-associated molecular pathway states (33). This indicates that TEPs may serve as effective liquid biopsy biosources that capture information on tumor presence and type. In extensive pan-cancer studies, platelet RNA analysis has identified 18 tumor types and inferred the primary tumor site, indicating that the unique role of TEPs extends beyond merely participating in tumor biological processes; they also act as a peripheral molecular mirror reflecting systemic tumor activity (34).

Furthermore, TEPs can carry specific tumor-associated non-coding RNAs. In patients with non-small cell lung cancer (NSCLC), abnormal expression of lncRNAs such as MAGI2-AS3, ZFAS1, linc-GTF2H2-1, RP3-466P17.2, lnc-ST8SIA4-12, STARD4-AS1, and ELOA-AS1 in TEPs suggests that TEPs can serve as stable carriers of lung cancer-associated lncRNA information (35–37).

However, changes in platelet RNAs are not restricted to cancer. The pan-cancer thromboSeq algorithm developed by In’t Veld et al. achieved 99% specificity in asymptomatic controls, while specificity decreased to 78% in symptomatic non-cancer controls with inflammatory diseases, cardiovascular diseases, or benign masses (34). Additionally, Karp et al. suggested that part of the TEP RNA signal may be associated with platelet activation and RNAs originating from inflammatory cells such as monocytes and lymphocytes (7). These findings indicate that non-malignant conditions may also affect platelet RNA profiles and increase false-positive results. Therefore, studies that utilize only healthy controls may overestimate the tumor specificity of TEP RNA biomarkers in real-world differential-diagnostic contexts.

Future studies should therefore include not only healthy controls but also clinically relevant non-cancer disease controls, such as inflammatory disorders, cardiovascular diseases, and benign masses, while carefully controlling for platelet activation and non-platelet RNA contamination.

### 3.2 TEPs for dynamic tumor monitoring

Another distinctive role of TEPs is their ability to reflect dynamic changes in tumor burden and treatment response. The RNA and protein profiles of normal platelets primarily relate to platelet production, aging, and physiological activation, whereas the molecular profiles of TEPs change in response to tumor resection, therapeutic intervention, recurrence, and disease progression.

A small prospective follow-up study on nasopharyngeal carcinoma (NPC) demonstrated that TEP miR-18a-3p expression level decreased during chemotherapy, exhibiting dynamic fluctuations that were broadly concordant with the levels of circulating EBV DNA (38).

In glioblastoma research, Sol et al. reported that TEP RNAs can be utilized for brain tumor detection, with the TEP score decreasing after tumor resection and increasing again during true progression. This dynamic change aids in distinguishing true tumor progression from pseudoprogression, suggesting that TEPs have potential value for diagnosis and treatment follow-up (39). Importantly, this study demonstrated that the RNA signal intensity of TEPs can change with tumor burden. This contrasts with normal platelets or transiently activated platelets, which generally do not reflect differences among tumor resection, pseudoprogression, and true progression.

Evidence from prostate cancer further supports the dynamic nature of tumor-associated platelet RNA cargo. Dudiki et al. detected the prostate cancer-associated lncRNA PCA3 in platelets of patients but not in healthy donors, and almost all patients from the same cohort became platelet PCA3-negative two months after radical prostatectomy (25). These findings indicate that tumor-associated platelet RNA signals can decrease following removal of the primary tumor. In the context of immunotherapy, TEP-derived PD-L1 mRNA in NSCLC patients has been proposed as a potential biomarker for evaluating clinical response and the likelihood of benefit following immunotherapy (40).

Overall, these studies indicate that TEP molecular profiles can change in parallel with tumor burden, treatment response, and disease progression. This dynamic responsiveness supports the potential of TEPs as biomarkers for monitoring therapeutic efficacy and detecting disease recurrence or progression.

### 3.3 TEPs regulate tumor progression

In addition to serving as carriers of tumor information, some TEPs or their derived vesicles can transfer RNA cargo to tumor cells, thereby directly altering tumor cell behavior (**Figure 1**). Compared with normal platelets, the RNA or protein cargo of TEPs is influenced by tumors, which may endow them with enhanced tumor-related functions.

In colorectal cancer (CRC), TIMP1 mRNA has been reported to be elevated in TEPs from CRC patients and can be transported into CRC cells by platelets, thereby promoting tumor growth (41) (**Figure 1**). In breast cancer, platelet TPM3 mRNA levels are increased in patients and are associated with metastasis. Further studies have shown that platelets can deliver TPM3 mRNA to breast cancer cells via microvesicles, enhancing their migratory phenotype (42) (**Figure 1**). This suggests that tumor-associated platelet RNAs may not only passively reflect tumor status but may also influence the metastatic capacity of tumor cells through vesicular transport.

Another direct functional example is platelet miR-223, whose expression is upregulated in patients with NSCLC relative to healthy control platelets. Platelet-derived microvesicles transfer miR-223 into lung cancer cells, where it associates with the RNA-induced silencing complex (RISC) and suppresses translation of the tumor suppressor EPB41L3, thereby reducing EPB41L3 protein expression and enhancing tumor-cell invasion (43).

Using breast cancer patient specimens, cellular co-culture systems, and murine models, Guha et al. demonstrated that TEPs directly engage breast cancer stem cells (BCSCs) through the interaction of platelet P-selectin with PSGL-1 on BCSCs, thereby activating WNT–FRIZZLED signaling and promoting β-catenin stabilization and nuclear translocation. Nuclear β-catenin subsequently reinforces the stem-like state by upregulating NANOG, OCT4, and SOX2, while simultaneously driving epithelial–mesenchymal transition through the induction of TWIST, SNAIL, and VIMENTIN and suppression of E-cadherin. This signaling program further stimulates VEGF production and autocrine VEGFR2 activation, establishing a WNT–β-catenin–VEGF–VEGFR2 feed-forward axis. Collectively, this network enhances BCSC clonogenicity, chemoresistance, invasiveness, angiogenic potential, and pulmonary metastatic capacity (44) (**Figure 1**). These findings support a direct role for TEP-mediated reprogramming of cancer stem cells in breast cancer progression.

Together, these studies indicate that TEPs are not only passive carriers of tumor information but may also actively deliver functional RNA and signaling cues that promote tumor growth, metastasis, and stem-like phenotypes.

### 3.4 TEP-mediated remodeling of the tumor microenvironment

The distinctive role of TEPs lies in tumor-induced changes in their molecular cargo, release patterns, and target-cell interactions, enabling them to exert functional effects within the tumor microenvironment that differ from those of normal platelets (45, 46) (**Figure 1**).

Within the tumor vascular microenvironment, TEPs secrete factors such as VEGF, EGF, bFGF, PDGF-B, and angiopoietins, which directly stimulate endothelial cell proliferation and tube formation. In glioblastoma (GBM), platelet VEGF levels are elevated in patients compared to healthy controls. Furthermore, stimulation with ADP or thrombin induces markedly augmented VEGF release from patient platelets, whereas endostatin secretion fails to increase correspondingly. Collectively, these observations indicate that TEPs isolated from GBM patients undergo a functional shift toward promoting tumor angiogenesis (46) (**Figure 1**). Through the PDGF-B/PDGFRβ signaling axis, TEPs recruit pericytes and smooth muscle cells to stabilize nascent blood vessels (47, 48). Simultaneously, TEPs facilitate the recruitment of tumor-associated macrophages (TAMs), further amplifying the release of matrix metalloproteinases (MMPs) and pro-angiogenic cytokines (49, 50) (**Figure 1**).

At the immunological level, platelet coating confers a platelet-mimetic MHC-I phenotype on circulating tumor cells (CTCs) (51). Surface GARP levels on TEPs are elevated in patients with melanoma compared with healthy platelets, and GARP–TGF-β signaling suppress NK cells and cytotoxic T lymphocytes (CTLs), while promoting the generation of regulatory T cells, myeloid-derived suppressor cells (MDSCs), and M2-polarized TAMs (52) (**Figure 1**). Collectively, these events skew the TME toward an immune-tolerant state.

In addition, tumor education can shift the intrinsic matrix- and vasculature-regulating functions of platelets toward a more prometastatic phenotype. Although MMP-2, MMP-9, TSP-1, ADP, ATP, TXA_2_, and LPA are not unique to TEPs, exposure to tumors modifies the molecular composition and functional state of platelets, thereby enhancing their ability to support tumor dissemination (6). In this tumor-conditioned state, MMP-2, MMP-9, and TSP-1 contribute to ECM remodeling, while ADP, ATP, TXA_2_, and LPA increase endothelial permeability and promote transendothelial migration of tumor cells, collectively creating a microenvironment more permissive to invasion and metastatic colonization.

Despite the diversity of TEP-mediated effects, several signaling networks appear to converge on common biological outcomes. P-selectin/PSGL-1–WNT/β-catenin and VEGF/VEGFR2 signaling promote stemness, EMT, and angiogenesis; PDGF-B/PDGFRβ contributes to vascular stabilization; GARP–TGF-β signaling promotes immune suppression; while MMP-associated extracellular-matrix remodeling facilitates invasion and metastatic dissemination. Thus, tumor education modifies platelet cargo and signaling capacity through distinct upstream mechanisms that ultimately converge on angiogenesis, immune evasion, EMT, invasion, and metastatic colonisztion.

Taken together, TEPs can remodel the tumor microenvironment by coordinating responses related to angiogenesis, stroma, immunity, and the extracellular matrix. Future studies can explore whether selectively disrupting TEP-mediated communication can remodel the tumor microenvironment without impairing the essential physiological functions of normal platelets.

## 4 Non-coding RNAs in tumor-educated platelets

In the TEP-mediated regulation of tumor progression, ncRNAs serve as key functional mediators. The primary ncRNA species include miRNAs, circRNAs, and lncRNAs, all of which have important regulatory functions and considerable translational potential in cancer diagnosis, prognostic assessment, and therapeutic development. By shaping platelet–tumor communication, these ncRNAs may contribute to tumor cell proliferation, invasion, metastasis, immune modulation, and treatment response. The following sections discuss the roles of platelet-associated miRNAs, circRNAs, and lncRNAs in cancer, with emphasis on their biological functions and clinical relevance (53, 54).

### 4.1 Platelet miRNAs in cancer

MiRNAs are a class of non-coding RNAs, typically ranging from 18 to 25 nucleotides in length, and primarily regulate gene expression by inducing the degradation of target mRNAs or repressing their translation (55). Although platelets lack nuclei, they inherit pre-miRNAs, mature miRNAs, target mRNAs, and partial components involved in miRNA processing from megakaryocytes (56, 57). In the context of cancer, tumor-derived signals and the tumor microenvironment selectively remodel the platelet miRNA profile, resulting in characteristic expression patterns that vary across different cancer types. These miRNAs have the potential to regulate tumor cell proliferation, migration, invasion, and other malignant processes.

Direct experimental evidence indicates that tumor cells can induce remodeling of platelet miRNA profiles, particularly in pancreatic cancer. Díaz-Blancas et al. showed that the platelet miRNA profiles of pancreatic cancer patients differed from those of healthy controls. Furthermore, blood-derived and pancreatic juice-derived platelets from the same patients displayed distinct miRNA patterns, suggesting that the local tumor microenvironment may shape heterogeneous platelet subpopulations. Utilizing a BxPC-3 pancreatic cancer cell–platelet co-culture model, they validated tumor-induced alterations in platelet miRNAs, including miR-711 and miR-600. Functionally, exposure to platelets enhanced BxPC-3 cell proliferation, migration, colony formation, and the expression of stemness-related genes, including NANOG, OCT4 and SOX2 (58) (**Figure 2**).

**Figure 2:**
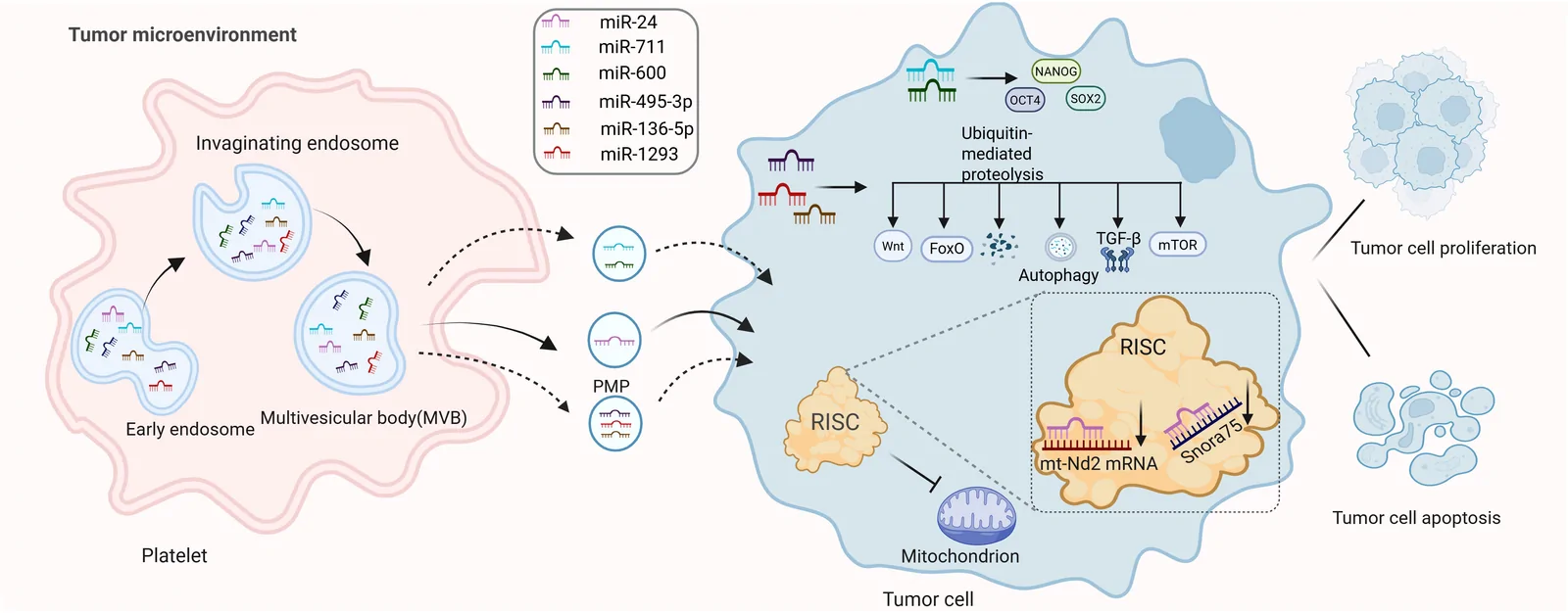
Proposed mechanisms of platelet-derived miRNAs in tumor cells. Platelet-derived microparticles (PMPs) may deliver miR-24, miR-711, miR-600, miR-495-3p, miR-136-5p, miR-1293 into tumor cells. miR-24 recruits mt-Nd2 mRNA and Snora75 to the RNA-induced silencing complex (RISC), thereby inducing mitochondrial dysfunction. miR-711 and miR-600 enhance the expression of stemness-related genes, including NANOG, OCT4 and SOX2. miR-495-3p, miR-136-5p, and miR-1293 revealed enrichment in the regulation of Wnt signaling, together with pathways involving FoxO, ubiquitin-mediated proteolysis, autophagy, TGF-β, and mTOR. These mechanisms regulate tumor cell proliferation and tumor cell apoptosis. Solid arrows indicate experimentally supported relationships, whereas dashed arrows indicate proposed or predicted relationships that have not yet been experimentally confirmed.

Tumor-associated remodeling of platelet miRNAs has also been observed in hepatocellular carcinoma (HCC). Zhu et al. identified 250 differentially expressed platelet miRNAs in patients with HCC and further validated the decreased levels of miR-495-3p and miR-136-5p and increased level of miR-1293 by qRT-PCR. Functional analysis of the predicted targets of these miRNAs revealed enrichment in the regulation of cellular catabolism, serine/threonine protein kinase activity, mRNA metabolism, and Wnt signaling, together with pathways involving FoxO, ubiquitin-mediated proteolysis, autophagy, TGF-β, and mTOR, all of which are closely linked to the progression of HCC (59) (**Figure 2**).

Furthermore, platelet-derived miR-24 has been shown to exert functional effects in Lewis lung carcinoma (LLC) cells. miR-24 functions by recruiting its target molecules, including mitochondrial mt-Nd2 mRNA and the non-coding RNA Snora75, to the RNA-induced silencing complex (RISC). In a miR-24-dependent manner, it suppresses the expression of these target RNAs and their corresponding proteins, thereby inducing mitochondrial dysfunction in tumor cells, triggering apoptosis, and inhibiting proliferation (53) (**Figure 2**).

In the context of hematologic malignancies, Girardot et al. reported that miR-28 was overexpressed in platelets from a subset of patients with myeloproliferative neoplasms, while remaining at relatively low levels in platelets from healthy subjects. This finding identifies miR-28 as a well-supported tumor-associated platelet miRNA in hematologic malignancies. Further mechanistic evidence showed that mutations such as JAK2 V617F, MPL W515, BCR-ABL, and constitutive STAT5 activation can induce miR-28 expression, whereas dominant-negative STAT5 reduces miR-28 expression. These data suggest a strong correlation between miR-28 upregulation and persistent JAK/STAT activation driven by malignant clones (60) (**Figure 2**).

Current studies have provided relatively detailed mechanistic insights into the role of platelet miRNAs in regulating malignant tumor phenotypes. Future research should further explore the potential of these miRNAs as therapeutically actionable targets.

### 4.2 Platelet circRNAs in cancer

CircRNAs represent a class of non-coding RNAs characterized by a covalently closed circular structure, which is formed through the back-splicing of pre-mRNAs. Due to the absence of free 5′ and 3′ termini, circRNAs exhibit significant resistance to degradation by exonucleases, resulting in high stability and enrichment in platelets (61, 62). In the tumor microenvironment, the circRNA profile of platelets is shaped by multiple processes, including RNA loading from megakaryocytes, uptake of tumor-derived extracellular vesicles, and tumor-associated splicing programs (28). Distinct tumor types produce divergent expression patterns of platelet circRNAs, making these transcripts promising potential biomarkers for tracing tumor origin, staging, and biological status (63). Structurally, these circRNAs mainly include exonic circRNAs derived from host-gene exons, along with smaller subsets of exon-intron circRNAs, intronic circRNAs, tricRNAs, and fusion-circRNAs resulting from cancer-associated chromosomal translocations (64, 65).

In addition to their biomarker potential, some platelet-associated circRNAs may influence tumor cell proliferation, invasion, and metastasis through miRNA-related, RNA-binding-protein, and downstream signaling networks; however, direct TEP-specific functional evidence remains limited.

D’Ambrosi et al. identified 411 differentially expressed circRNAs in platelets from patients with NSCLC. Among these, circNRIP1 was significantly downregulated in an independent RT-qPCR validation cohort and showed a stronger association with advanced-stage NSCLC (63). Functionally, circNRIP1 has been implicated in cancer cell proliferation, migration, invasion, and chemoresistance across several tumor types, potentially through miRNA-sponging activity or AKT/mTOR-related pathways. The same research group later integrated platelet-derived circRNAs with mRNA profiles.

Razzaghi et al. reported that hsa_circ_0004771 and hsa_circ_0019120 are elevated in platelets from patients with CRC (66). Mechanistically, circNRIP1 may exert a more direct biological association with CRC progression. circNRIP1 participates in 5-FU resistance by regulating the miR-653/ZEB2 axis (67) (**Figure 3**). However, it remains to be validated whether platelet circNRIP1 can be transferred into CRC cells to modulate the miR-653/ZEB2 axis.

**Figure 3:**
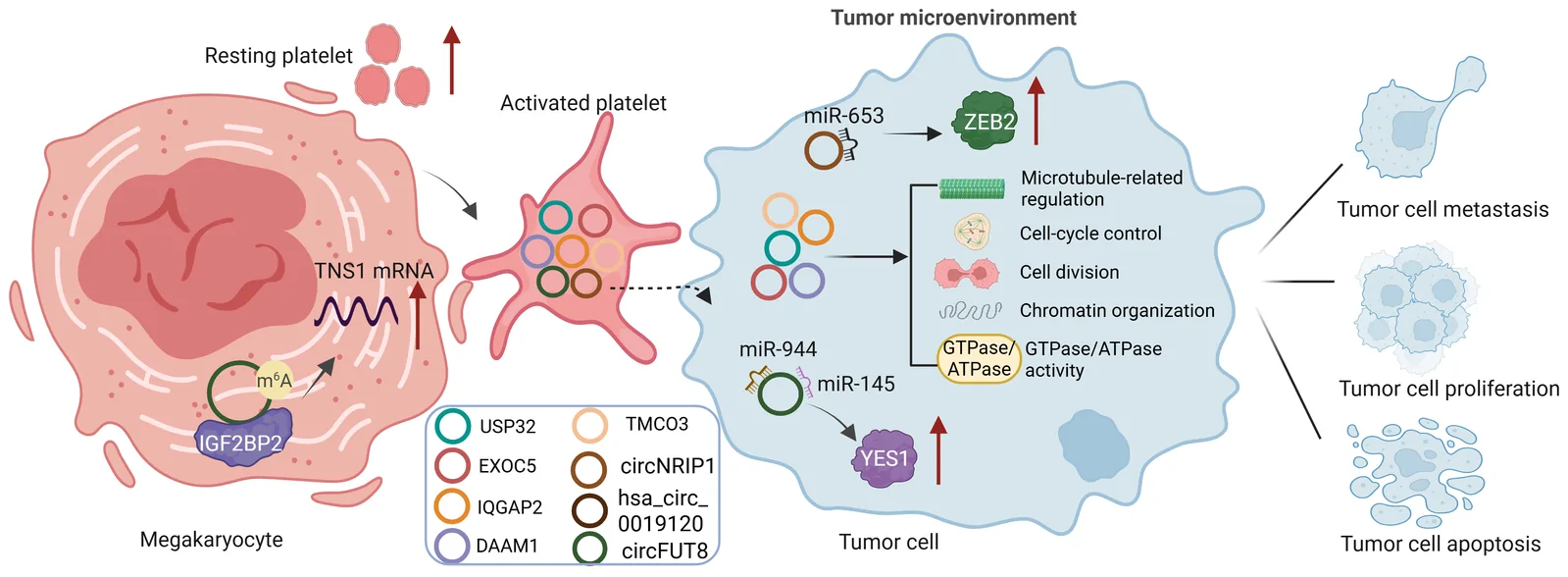
Proposed mechanisms of platelet-derived circRNAs in tumor cells. During megakaryocyte differentiation and maturation, circFUT8 binds to IGF2BP2 in an m^6^A-dependent manner and stabilizes TNS1 mRNA, thereby contributing to thrombopoiesis and platelet production. Activated platelets contain USP32, EXOC5, IQGAP2, DAAM1, TMCO3, circNRIP1, circBTAF1, and circFUT8, and may transfer these circRNAs to tumor cells. circFUT8 can sponge miR-145 or modulate the miR-944/YES1 axis. USP32, EXOC5, IQGAP2, DAAM1, and TMCO3 are involved in cell-cycle control, cell division, chromatin organization, GTPase/ATPase activity, and microtubule-related regulation. circNRIP1 regulates the miR−653/ZEB2 axis. These mechanisms regulate tumor cell proliferation, tumor cell metastasis, and tumor cell apoptosis. Solid arrows indicate experimentally supported relationships, whereas dashed arrows indicate proposed or predicted relationships that have not yet been experimentally confirmed.

In gastroenteropancreatic neuroendocrine tumors (GEP-NETs), Campolo et al. identified 252 differentially expressed TEP circRNAs. After stringent false discovery rate (FDR) filtering, five markedly downregulated circRNAs were retained, corresponding to USP32, EXOC5, IQGAP2, DAAM1, and TMCO3. Functional enrichment analysis indicated that the genes associated with these circRNAs were mainly involved in cell-cycle control, cell division, chromatin organization, GTPase/ATPase activity, and microtubule-related regulation, suggesting that TEP circRNAs may capture biological processes linked to tumor proliferation and cytoskeletal remodeling (68) (**Figure 3**).

CircFUT8 exhibits elevated expression during the differentiation of human umbilical cord blood hematopoietic stem cells (hUCB-HSCs) into megakaryocytes. It binds to IGF2BP2 in an m^6^A-dependent manner, stabilizes TNS1 mRNA and thereby contributes to actin polymerization, megakaryocyte maturation, and platelet production (69). Additionally, circFUT8 expression is upregulated in platelets within the lung cancer microenvironment. Importantly, platelet-derived circFUT8 may be transferred to lung cancer cells and modulate metastasis, proliferation, and apoptosis by sponging miR-145 or regulating the miR-944/YES1 axis (69) (**Figure 3**).

Despite the increasing evidence supporting the potential of platelet circRNAs as biomarkers, their functional roles and underlying molecular mechanisms remain inadequately characterized. Therefore, future studies should extend beyond mere expression profiling to further elucidate how platelet circRNAs are generated, selectively enriched, transferred, and functionally engaged in platelet-tumor communication.

### 4.3 Platelet lncRNAs in cancer

LncRNAs generally refer to transcripts longer than 200 nucleotides that lack substantial protein-coding capacity (70). They can modulate transcript stability, translational efficiency, and cellular signaling networks by interacting with other RNAs or RNA-binding proteins (71, 72). Similar to miRNAs and circRNAs, platelet lncRNAs within the tumor microenvironment are RNA cargo shaped by a combination of megakaryocyte origin, platelet-intrinsic RNA metabolism, and tumor-associated signaling (21). Consequently, lncRNA expression profiles in TEPs may vary according to cancer type, tumor stage, and metastatic status. These profiles may reflect tumor subtypes and disease progression, while some platelet-associated lncRNAs may also have functional regulatory roles; however, direct TEP-specific mechanistic evidence remains limited.

Existing evidence suggests that alterations in platelet lncRNAs show distinct cancer-associated expression patterns. In NSCLC, MAGI2-AS3 and ZFAS1 are significantly downregulated in the TEPs of patients. Furthermore, MAGI2-AS3 is associated with TNM stage, lymph node metastasis, and distant metastasis, indicating that it possesses diagnostic value and may also reflect tumor progression (35). Mechanistically, MAGI2-AS3 has been shown to exert tumor-suppressive effects in NSCLC by functioning as a competing endogenous RNA, including regulation of the miR-629-5p/TXNIP and miR-374a/b-5p/CADM2 axes, thereby suppressing proliferation, migration, and invasion while promoting apoptosis (73). ZFAS1 can promote NSCLC progression through the miR-150-5p/HMGA2 axis, enhancing tumor-cell proliferation and invasion and reducing apoptosis (74). These tumor-cell mechanisms provide a potential biological basis for the association of altered MAGI2-AS3 and ZFAS1 profiles with tumor progression, but have not yet been directly demonstrated as TEP-mediated mechanisms (**Figure 4**).

**Figure 4:**
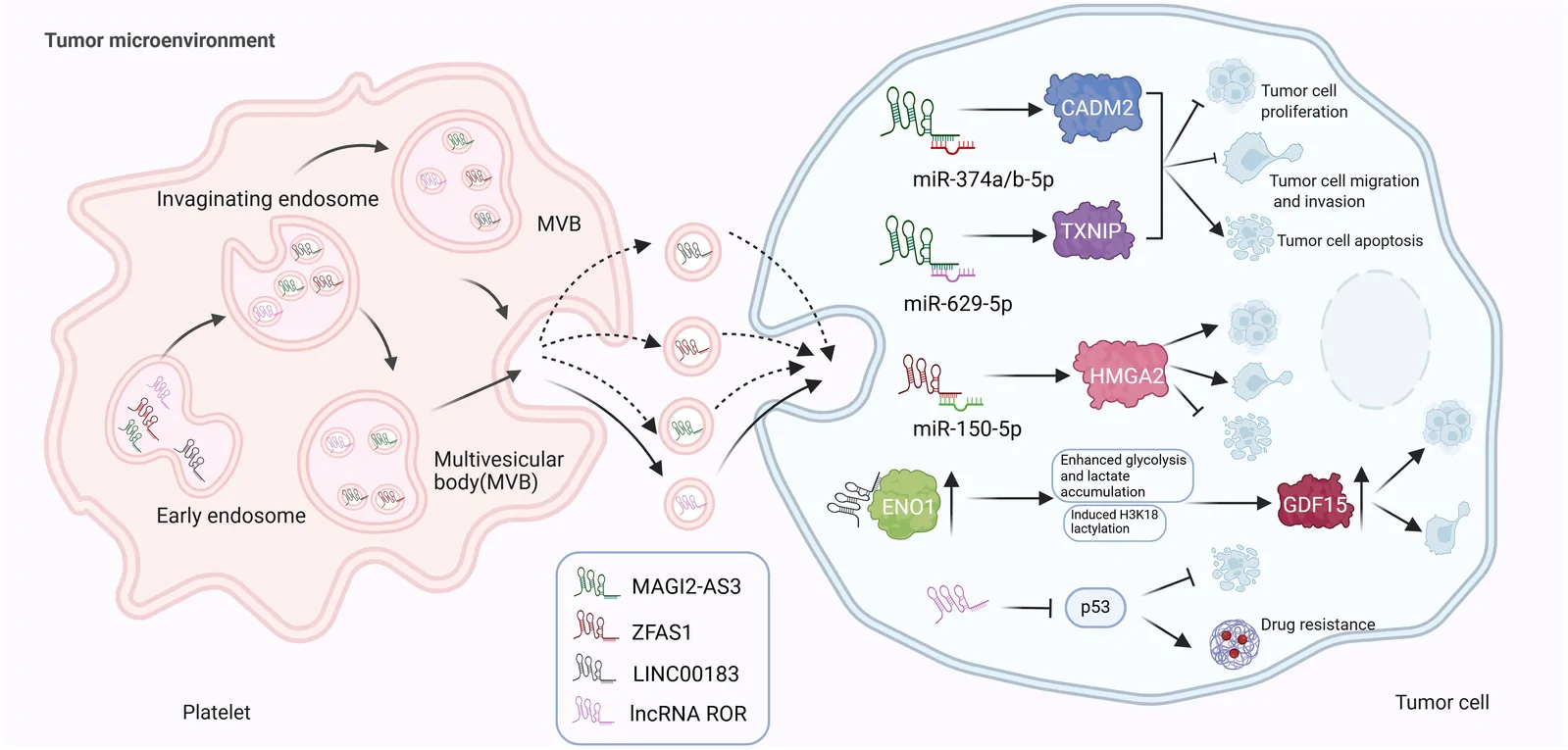
Proposed mechanisms of platelet lncRNAs in tumor cells. Platelet-derived microparticles (PMPs) may deliver MAGI2-AS3, ZFAS1, LINC00183, and lncRNA ROR into tumor cells. lncRNA ROR suppresses the p53 signaling pathway. MAGI2-AS3 regulates the miR-629-5p/TXNIP and miR-374a/b-5p/CADM2 axes. ZFAS1 regulates miR-150-5p/HMGA2 axis. LINC00183 bound to ENO1, prevented its degradation via the ubiquitin-proteasome pathway, enhanced glycolysis and lactate accumulation, induced H3K18 lactylation, and further promoted the transcriptional upregulation of GDF15. These mechanisms regulate tumor cell proliferation, tumor cell migration and invasion, tumor cell apoptosis, and drug resistance. Solid arrows indicate experimentally supported relationships, whereas dashed arrows indicate proposed or predicted relationships that have not yet been experimentally confirmed.

Additionally, LINC00183 in platelet-derived exosomes from CRC patients was significantly elevated and could be transferred to CRC cells. Subsequently, it bound to ENO1, prevented its degradation via the ubiquitin-proteasome pathway, enhanced glycolysis and lactate accumulation, induced H3K18 lactylation, and further promoted the transcriptional upregulation of GDF15, ultimately enhancing the proliferation, invasion, and metastasis of CRC cells (54) (**Figure 4**).

In patients with NPC, TEP lncRNA ROR was significantly downregulated in platelets, while no significant difference was observed in plasma lncRNA ROR levels. This finding supports the potential value of platelet-associated lncRNA ROR as a biomarker distinct from plasma ROR levels. Additionally, lncRNA ROR may interact with NPC tissues through vesicle-mediated transfer, promoting the proliferation and migration of nasopharyngeal cancer cells. It also inhibits tumor cell apoptosis and enhances resistance to chemotherapy by suppressing the p53 signaling pathway (**Figure 4**). This interaction is closely associated with patient prognosis (75). Together, these findings suggest that platelet-associated lncRNAs may serve both as cancer-associated molecular signatures and as functional regulators of tumor metabolism, metastasis, and therapeutic resistance.

Overall, platelet lncRNAs may link tumor-induced molecular remodeling of platelets to subsequent alterations in tumor behavior. Future studies should broaden the range of cancer types and lncRNA candidates to further elucidate the specific mechanisms through which lncRNAs influence tumor progression.

### 4.4 Comparison of three classes of platelet ncRNAs

Although miRNAs, circRNAs, and lncRNAs all exhibit promising potential as functional effector molecules and liquid biopsy biomarkers within TEPs, these three classes of ncRNAs differ in their current evidence base and translational maturity (32).

Among the three classes, miRNAs currently have the strongest mechanistic evidence. Studies have progressed from expression profiling and disease associations to molecular transfer, target validation, and demonstration of functional effects (53). In contrast, platelet circRNA research remains more strongly focused on biomarker discovery and validation (76). The covalently closed circular structure of circRNAs confers high stability and resistance to exonuclease-mediated degradation, making them particularly attractive candidates for liquid-biopsy applications (77). However, direct functional evidence linking specific platelet-associated circRNAs to tumor-cell phenotypes remains comparatively limited. Platelet-associated lncRNAs may provide complementary regulatory information related to cancer type, stage, and disease progression, but their TEP-specific functional mechanisms remain less well validated (35). Thus, the three ncRNA classes should not be considered equally mature from a translational perspective. miRNAs currently have the strongest mechanistic basis and may be suitable for further development as functional targets or components of multi-marker panels. CircRNAs may be particularly advantageous as stable liquid-biopsy biomarkers because of their structural stability and enrichment in platelets, whereas lncRNAs may provide additional biological and cancer-associated regulatory information but require further mechanistic validation.

In addition, the evidence base across all three ncRNA classes is constrained by small cohort sizes, heterogeneous platelet isolation methods, variation in sequencing and qRT-PCR platforms, inconsistent normalization strategies, and insufficient independent external validation32. Addressing these methodological limitations will be essential for determining the relative clinical utility of each ncRNA class (32).

## 5 Non-coding RNAs in tumor-educated platelets guiding cancer diagnosis and treatment

The unique expression profiles of ncRNAs in TEPs can serve as potential diagnostic and monitoring biomarkers for cancer (**Table 1**). Furthermore, these profiles may provide molecular targets and inform the development of novel RNA-based or platelet-associated therapeutic strategies. However, most current evidence remains at the discovery or preclinical stage. Therefore, further validation in large, independent, and clinically well-characterized cohorts is required before TEP ncRNAs can be implemented in routine cancer diagnosis or treatment.

**Table 1:** Characteristics, biological functions, and translational status of TEP ncRNA biomarkers in cancer.

Marker type	TEP biomarker	Cancer	Expression	Technique	Biological function	Evidence stage	Ref.
miRNAs	miR-223	LC	Up	qRT-PCR	miR−223 reduces EPB41L3 protein expression by binding to the 3′ UTR of tumor−suppressor gene EPB41L3 mRNA and inhibiting its translation, thereby promoting lung cancer cell invasion.	Preclinical	(43)
	miR-711	PC	Up	qRT-PCR	Tumor exposure alters platelet miR-711 and miR-600 levels; platelet exposure is associated with increased pancreatic cancer cell proliferation, migration, clonogenicity, and stemness-related gene expression.	Discovery	(58)
	miR-600		Up				
	miR-495-3p	HCC	Down	RNA-seq, qRT-PCR	Predicted-target enrichment implicated pathways involving cellular catabolism, Wnt, FoxO, ubiquitin-mediated proteolysis, autophagy, TGF-β, and mTOR.	Validation	(59)
	miR-136-5p		Down				
	miR-1293		Up				
	miR-34c-3P	NPC	Up	qRT-PCR	miR-34C-3P and miR-18a-5p regulate the expression of oncogenes and tumor−suppressor genes by participating in cell proliferation, differentiation, apoptosis, invasion, and metastasis.	Validation	(78)
	miR-18a-5p		Up				
	miR-28	MPN	Up	qRT-PCR	miR-28 targets the 3′ UTR of the thrombopoietin receptor MPL and inhibits its translation. It also regulates protein-coding genes involved in megakaryocyte differentiation, thereby contributing to the pathogenesis of MPNs.	Preclinical	(60)
	miR-199b-3p	LC	Down	RNA-seq, qRT-PCR	miR−199b−3p targets KTN1, which participates in cell adhesion, mitotic spindle regulation, migration and invasion and correlates with poor prognosis	Validation	(79)
	miR-24	LLC	Up	qRT-PCR	miR-24 recruits mt-Nd2 mRNA and Snora75 to RISC and suppresses their expression and associated protein production in an miR-24-dependent manner, thereby inducing mitochondrial dysfunction, apoptosis, and inhibition of tumor cell proliferation.	Preclinical	(53)
lncRNAs	lncRNA CCAT1	CRC	Up	qRT-PCR	–	Validation	(88)
	lncRNA HOTTIP		Up				
	AL355574.1	GC	Up	qRT-PCR	These lncRNAs affect prognosis and immunotherapy efficacy in patients with gastric cancer by influencing immune−related signaling pathways, including complement and coagulation cascades and ECM–receptor interactions, and by linking DNA methylation to the EMT process.	Discovery	(93)
	AL513123.1		Up				
	AC002401.4		Up				
	LINC01094		Up				
	AL356417.2		Up				
	LINC01697		Down				
	AC129507.1		Down				
	linc-GTF2H2-1	LC	Down	qRT-PCR	These lncRNAs influence tumor development, progression, and metastasis by participating in the regulation of gene expression.	Validation	(36)
	RP3-466P17.2		Down				
	lnc-ST8SIA4-12		Up				
	lncRNA ROR	NPC	Down	qRT-PCR	lncRNA ROR promotes the proliferation, metastasis, and chemoresistance of nasopharyngeal carcinoma cells by inhibiting the p53 signaling pathway.	Validation	(75)
	MAGI2-AS3	NSCLC	Down	qRT-PCR, ARMS-PCR	MAGI2-AS3 and ZFAS1 are closely associated with TNM stage, lymph-node metastasis, and distant metastasis.	Validation	(35)
	ZFAS1		Down				
	LNCAROD	CRC	Up	RNA-seq, qRT-PCR	LNCAROD modulates downstream signaling pathways by forming a ternary complex with HSPA1A and YBX1 and being stabilized by m^6^A methylation, thereby driving malignant tumor cell proliferation and progression.	Validation	(9)
	SNHG20		Up		SNHG20 promotes cancer cell growth, migration, and invasion by regulating the expression of the cell-cycle-related genes p21 and cyclin A1.		
	LINC00534		Up		–		
	TSPOAP-AS1		Up		–		
lncRNAs	STARD4-AS1	NSCLC	Up	qRT-PCR	STARD4-AS1 exerts cis-regulatory effects on the neighboring STARD4 gene, remodels cholesterol metabolism in tumor cells, and establishes a systemic lipid metabolic microenvironment conducive to metastasis through the circulation, thereby accelerating tumor progression.	Validation	(37)
	ELOA-AS1		Up		ELOA-AS1 directly activates the EMT signaling pathway, thereby promoting tumor cell proliferation, invasion, and metastasis.		
	LINC00183	CRC	Up	qRT-PCR	LINC00183 promotes glycolysis and the malignant progression of colorectal cancer cells through the ENO1/H3K18la/GDF15 axis.	Preclinical	(54)
circRNAs	circNRIP1	NSCLC	Down	RNA-Seq, qRT-PCR	–	Validation	(63)
	circUSP32	GEP-NET	Down	RNA-Seq	–	Discovery	(68)
	circEXOC5		Down				
	circIQGAP2		Down				
	circDAAM1		Down				
	circTMCO3		Down				
	circFUT8	LC	Up	NanoString nCounter	circFUT8 modulates lung cancer cell proliferation, invasion, and apoptosis by sponging miR-145 or regulating the miR-944/YES1 axis.	Discovery	(69)
	hsa_circ_0004771	CRC	Up	qRT-PCR	–	Validation	(66)
	hsa_circ_0019120						
	hsa_circ_0061274	LC	Down	RNA-seq, qRT-PCR	–	Validation	(84)

PC, pancreatic cancer; HCC, hepatocellular carcinoma; NPC, nasopharyngeal carcinoma; MPN, myeloproliferative neoplasms; TC, thyroid cancer; OC, ovarian cancer; CRC, colorectal cancer; GC, gastric cancer; NSCLC, non-small cell lung cancer; GEP-NET, gastroenteropancreatic neuroendocrine tumor; LC, lung cancer; EMT, epithelial-mesenchymal transition; ECM, extracellular matrix; LLC, Lewis lung carcinoma; Discovery, initial expression/profiling evidence; Validation, candidate confirmed in an independent or targeted clinical cohort; Preclinical, functional/mechanistic evidence in cellular or animal models.

### 5.1 Platelet miRNAs guiding the diagnosis and therapy for tumors

Platelet-derived miRNAs have the advantages of easy sample acquisition, high RNA stability, reproducible detection, and minimal interference from plasma free RNA degradation. During tumorigenesis, tumor cells and their microenvironment reshape platelet RNA cargo, forming tumor-associated diagnostic signatures in platelet miRNA profiles. A study on pancreatic cancer confirmed that exposure to tumor cells induces dysregulation of platelet miRNA profiles, suggesting that TEP miRNAs reflect the status of tumor-platelet interaction.51 Furthermore, research on nasopharyngeal carcinoma showed that miR-34c-3p and miR-18a-5p were significantly elevated in patient-derived TEPs, with no corresponding changes in plasma, demonstrating promising diagnostic performance and indicating that platelet miRNAs can provide more tumor-relevant information for liquid biopsies (78).

In hepatocellular carcinoma, RNA-seq identified multiple differentially expressed TEP miRNAs, supporting the potential of platelet miRNA profiles for liver cancer screening and auxiliary diagnosis (59). A study on lung adenocarcinoma showed significant downregulation of miR-199b-3p in patient platelets, effectively distinguishing lung adenocarcinoma from benign pulmonary nodules, thereby confirming the value of platelet miRNAs in differentiating between benign and malignant lesions (79). In summary, the clinical significance of TEP miRNAs should be regarded as cancer-type-specific molecular signatures shaped by tumor-platelet crosstalk. Their potential applications include early tumor screening, differentiation of benign and malignant lesions, monitoring therapeutic responses, and the development of multi-marker diagnostic models in conjunction with traditional serological indicators.

Current evidence for therapeutically modulating platelet miRNAs remains preclinical but provides proof of principle. Cancer-related miRNAs can be classified as either tumor-suppressive or oncogenic, with some exhibiting dual functions. Technological advancements have facilitated the development of miRNA mimics and inhibitors, which can modulate the expression of specific miRNAs for targeted cancer treatment (**Figure 5**). In preclinical animal models of various cancers, including lung and breast cancer, the introduction of tumor-suppressive miRNA mimics has resulted in increased expression and inhibited tumor growth, potentially enhancing immune responses (80, 81). Similarly, in models of lymphoma and leukemia, the application of miRNA inhibitors to suppress oncogenic miRNAs has shown significant anti-tumor effects (82, 83). However, direct therapeutic targeting of platelet-derived or TEP-specific miRNAs remains at an early stage, and further studies are needed to establish delivery efficiency, specificity, safety, and clinical relevance.

**Figure 5:**
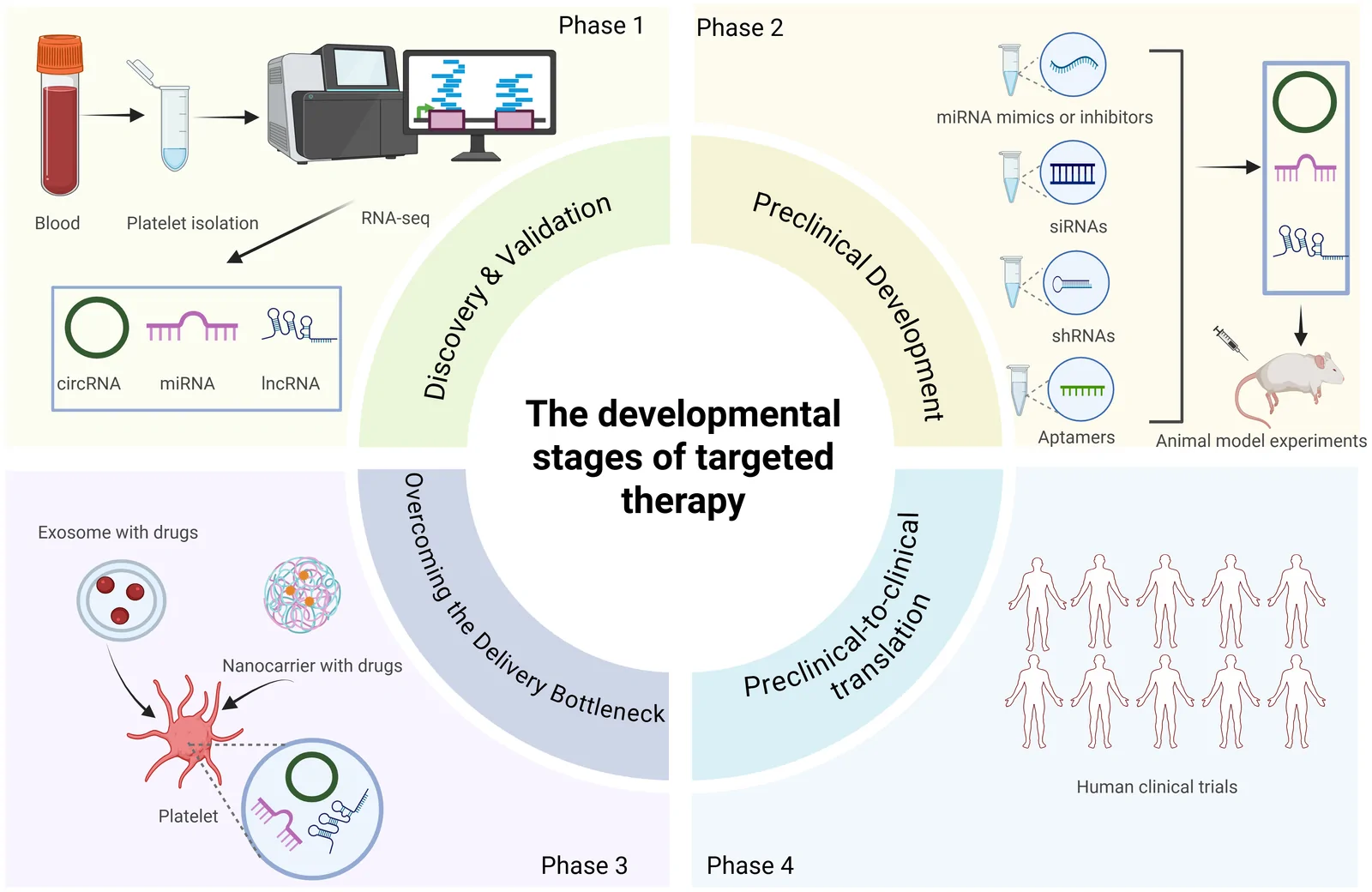
Translational development of TEP-associated ncRNAs for cancer diagnosis and therapy. Phase 1, Discovery and validation: platelet isolation, high-throughput RNA sequencing and bioinformatic analysis are used to identify tumor-associated ncRNA signatures, including miRNAs, circRNAs, and lncRNAs, in tumor-educated platelets (TEPs) across different cancer types. Phase 2, Preclinical development: candidate ncRNAs are functionally evaluated using experimental models, and therapeutic strategies such as miRNA mimics/inhibitors, siRNAs, shRNAs, antisense oligonucleotides, and aptamers may be explored to modulate cancer-associated ncRNA pathways. Phase 3, Overcoming the delivery bottleneck: engineered exosomes, nanocarriers and platelet-based delivery approaches may improve RNA delivery while reducing off-target effects and systemic toxicity. Phase 4, Preclinical-to-clinical translation: promising diagnostic or therapeutic candidates require validation in animal models, followed by carefully designed human clinical studies. At present, most TEP-associated ncRNA applications remain at the discovery or preclinical stage, and further validation is required before routine clinical use.

### 5.2 Platelet circRNAs guiding the diagnosis and therapy for tumors

The expression profiles of circRNAs in platelets from cancer patients differ significantly from those of non-cancer controls, with dynamic changes observed across various tumor stages and metastatic states. This tumor-specific expression pattern provides a valuable source of potential biomarkers for early diagnosis, disease monitoring, and prognostic evaluation.

Researchers conducted RNA sequencing analysis on TEP samples from 10 patients diagnosed with gastroenteropancreatic neuroendocrine tumors at baseline, alongside platelet samples from 5 patients with non-malignant endocrine diseases. This analysis identified five circRNAs, USP32, EXOC5, IQGAP2, DAAM1, and TMCO3, that were significantly downregulated and may be implicated in the development and progression of various cancers (68).

Notably, in lung adenocarcinoma, the quantity and abundance of circRNAs in platelets are significantly elevated compared with those in plasma exosomes. Additionally, hsa_circ_0061274 is markedly downregulated in the platelets of patients and can effectively differentiate lung adenocarcinoma from healthy controls and benign pulmonary nodules, indicating its potential clinical utility in distinguishing between benign and malignant pulmonary nodules (84).

Direct therapeutic evidence for platelet circRNAs is currently lacking. Nevertheless, the feasibility of experimentally manipulating circRNAs has been demonstrated. By inhibiting or upregulating specific cancer-associated circRNAs, tumor growth, metastasis, and response to treatment can be significantly affected. RNA interference (RNAi) technology is commonly employed to target circRNAs. Small interfering RNAs (siRNAs) or short hairpin RNAs (shRNAs) can be designed to target specific sequences of circRNAs, thereby facilitating their degradation and inhibiting their tumor-promoting functions (85, 86) (**Figure 5**). Similarly, antisense oligonucleotides can be utilized to specifically regulate circRNAs, modifying their biological activity (87).

Future studies should strengthen research on direct therapeutic targeting of platelet circRNAs and confirm platelet-specific delivery, target selectivity, safety, and therapeutic efficacy.

### 5.3 Platelet lncRNAs guiding the diagnosis and therapy for tumors

Recent research on platelet lncRNAs has led to growing interest in tumor diagnosis. In CRC, a study involving quantitative qRT-PCR on 75 CRC patients and 42 healthy control subjects revealed that the expression levels of TEP lncRNAs CCAT1 and HOTTIP were significantly upregulated in CRC patients compared with healthy individuals (88). Additionally, another study on CRC identified four lncRNAs—LNCAROD, SNHG20, LINC00534, and TSPOAP-AS1—that were upregulated in both TEPs and serum (9). These specifically expressed lncRNAs represent potential biomarkers for the diagnosis of colorectal cancer.

In NPC, the level of platelet lncRNA ROR before treatment was found to be significantly lower in patients with locally advanced NPC compared with the control group, suggesting that platelet lncRNA ROR could serve as a valuable diagnostic predictor in locally advanced NPC (75).

With the growing understanding of platelet lncRNAs, extensive exploration has been conducted in the field of tumor treatment. However, therapeutic targeting of platelet-derived or TEP-specific lncRNAs remains at an early stage. In addition to RNAi and antisense oligonucleotide-mediated targeted therapies, aptamer technology has emerged as a novel strategy for targeting cancer-associated lncRNA pathways. Aptamers are short single-stranded nucleic acids that are specifically screened to recognize and bind to target molecules (89) (**Figure 5**). In clinical applications, aptamers offer distinct advantages over traditional protein-based antibodies, including smaller size for improved tissue penetration, lower immunogenicity, higher stability, and ease of chemical modification. These diverse technical strategies for modulating lncRNA-related pathways may provide future opportunities for cancer treatment, but further validation is required to establish platelet-specific delivery, target selectivity, safety, and therapeutic efficacy. These studies provide a preclinical foundation for therapeutic targeting of platelet lncRNAs and highlight their potential for future clinical translation.

Overall, several ncRNA-modulation approaches, including ASOs, shRNAs, and miRNA mimics/inhibitors, have demonstrated feasibility in experimental systems. However, there is currently no established platform that enables selective *in vivo* targeting and therapeutic modulation of endogenous ncRNAs specifically in TEPs. Platelet-based carriers have also been explored for nucleic-acid delivery, but their application to TEP-specific ncRNA modulation remains prospective.

### 5.4 Current limitations and translational challenges

Despite the growing interest in TEP ncRNAs, several important challenges must be addressed before clinical translation. First, many studies are based on relatively small cohorts and lack large, multicenter validation. This limits the generalizability of reported ncRNA signatures across populations, cancer stages, and treatment backgrounds (76). Second, platelet isolation and RNA extraction protocols vary between studies, which may affect RNA yield, purity, and reproducibility (90). Contamination by leukocytes, plasma extracellular vesicles, or circulating tumor-derived nucleic acids may also confound interpretation if not carefully controlled (91).

Third, analytical platforms differ across studies, including RNA sequencing, microarray analysis, NanoString, qRT-PCR, and bioinformatic prediction methods (76, 79). These methodological differences make direct comparison between studies difficult. Fourth, many proposed biomarkers lack external validation, longitudinal assessment, and comparison with existing clinical biomarkers (92). Therefore, future studies should include standardized pre-analytical procedures, independent validation cohorts, diagnostic performance metrics such as sensitivity, specificity, and AUC, and evaluation of whether TEP ncRNA signatures provide added value beyond established biomarkers.

Finally, although ncRNA-based therapeutic strategies are promising, direct targeting of TEP ncRNAs remains largely preclinical (54). Major barriers include platelet-specific delivery, off-target effects, immune activation, RNA stability, dose control, and safety. Future research should distinguish clearly between cancer-associated ncRNA therapy in general and therapeutic modulation of platelet-derived or TEP-specific ncRNAs.

Taken together, these limitations indicate that the clinical translation of TEP ncRNAs will require a stepwise development process (**Figure 5**). Candidate ncRNAs should first undergo rigorous discovery and validation, followed by functional and preclinical evaluations to determine whether their modulation yields significant antitumor effects. Subsequent development should focus on overcoming the delivery bottlenecks by enhancing platelet or TEP selectivity while minimizing off-target effects, immune activation, and systemic toxicity. Engineered extracellular vesicles, nanocarriers, and platelet-based delivery systems may offer potential solutions to these challenges. Only after efficacy, target selectivity, pharmacological control, and safety have been sufficiently established should promising candidates progress toward clinical evaluation.

## 6 Conclusion and future perspectives

The complex interactions between tumors and platelets underscore the important roles of tumor-educated platelet ncRNAs in cancer. These interactions reshape platelet RNA profiles and generate tumor-associated molecular signatures with potential diagnostic and therapeutic relevance.

For diagnosis, high-throughput sequencing and bioinformatics enable comprehensive screening of platelet ncRNAs across tumor types and stages, supporting the discovery of candidate biomarkers for early detection, disease monitoring, and prognosis assessment. For instance, among currently reported candidate biomarkers, TEP miR-34c-3p and miR-18a-5p exhibit relatively favorable diagnostic performance in NPC. Platelet circNRIP1 has been validated in independent cohorts of patients with NSCLC, while platelet lncRNA ROR may serve as a potential adjunct to EBV DNA testing. However, most current evidence remains at the discovery or early validation stage. Large multicenter cohorts are required to confirm whether TEP ncRNA signatures are reproducible across cancer types, stages, and treatment settings. Standardized platelet isolation, RNA extraction and, data normalization procedures are also essential to improve comparability between studies. Longitudinal studies are further needed to determine whether changes in TEP ncRNA profiles can reliably reflect tumor burden, treatment response, recurrence, or resistance.

For treatment, targeting platelet ncRNAs holds promise, but direct therapeutic application of TEP ncRNAs remains at an early stage. Strategies such as miRNA mimics or inhibitors, RNA interference, antisense oligonucleotides, engineered exosomes, and nanocarrier-based delivery systems may provide future opportunities for modulating cancer-associated ncRNA pathways. Nevertheless, major challenges remain, including platelet-specific delivery, off-target effects, immune activation, RNA stability, dose control, and safety. Concepts such as time-dependent RNA delivery or combination RNA-based therapy are interesting but remain exploratory and require stronger experimental evidence before clinical translation.

Future research should move beyond descriptive profiling toward mechanistic and clinically validated studies. Importantly, many conventional experimental models of platelet–tumor interactions use naïve platelets from healthy donors or healthy mice rather than authentic TEPs. These models may not fully replicate the biological characteristics and functions of TEPs within the tumor microenvironment. Therefore, more physiologically relevant systems using TEPs directly isolated from tumor-bearing hosts or cancer patients will be critical for validating existing findings and defining the genuine regulatory mechanisms of platelet ncRNAs in cancer.

Overall, TEP ncRNAs represent a promising but still developing field. Their future clinical value will depend on robust validation, methodological standardization, mechanistic clarification, and careful distinction between general cancer-associated ncRNA biology and TEP-specific ncRNA functions.
